# Solid-state random microlasers fabricated via femtosecond laser writing

**DOI:** 10.1038/s41598-018-31966-6

**Published:** 2018-09-10

**Authors:** Nathália B. Tomazio, Lucas F. Sciuti, Gustavo F. B. de Almeida, Leonardo De Boni, Cleber R. Mendonca

**Affiliations:** São Carlos Institute of Physics, University of São Paulo, PO Box 369, 13560-970 São Carlos, SP Brazil

## Abstract

Here we demonstrate resonant random lasing in Rhodamine B-doped polymeric microstructures fabricated by means of femtosecond laser writing via two-photon polymerization. To the best of our knowledge, this is the first demonstration of random lasing action in on-chip microdevices. Their feedback mechanism relies on diffuse reflections at the structure sidewall surfaces, which is known as spatially localized feedback since the scattering centers lie over the edges of the gain medium. By exciting the structures with a pulsed laser at 532 nm, a multimode emission with randomly distributed narrow peaks was observed, in accordance with the random nature of the feedback mechanism. Interestingly, their lasing threshold was found to be on the order of tens of nanojoules, which is comparable to what had been achieved for usual microcavities, thereby demonstrating the potentiality of these devices as solid-state lasers for integrated optics applications.

## Introduction

Organic dyes have been extensively used in the development of laser systems because they stand for wavelength tunability, high lasing efficiency and both, continuous and pulsed operation with great beam quality^1–4^. On the other hand, dye lasers rely on complex setups that easily leads to beam misalignment, which combined with the dyes toxicity, has made them less competitive to semiconductor lasers^5^. A strategy to overcome these setbacks has been the use of polymers as a host matrix to dyes, creating solid-state dye lasers^6–10^. Besides its neat solid architecture, this type of lasers benefits from the low cost and processing flexibility of polymers, which broadens the range of cavity designs to be made^11^.

Different architectures have been realized to accomplish compact organic laser devices, such as vertical external cavity surface emitting lasers^12^, whispering gallery cavity lasers^7,8,13^ and distributed feedback Bragg lasers^14,15^. Taking advantage of the multiple scattering of light in disordered media has also been a way of providing positive feedback for lasing^16,17^. The lasers supplied by this feedback mechanism are known as random lasers (RL) and have been realized in polymeric platforms by either integrating scattering centers to the sample^18^ or taking advantage of irregularities that produce a random variation of the refractive index throughout the active medium^19^. Despite their multimode and omnidirectional operation, polymeric RLs hold great promise for applications in a variety of fields including biology sensing, encoding of information and display technology^5^.

Lasing action in RLs is usually achieved with a spatially distributed feedback^20–22^, where scattering elements are spread over the active medium as a whole, yet some studies have shown that positive feedback may be provided from diffuse reflection at a scattering surface^23,24^. This approach was first introduced in the early seventies, where one of the mirrors of a Fabry-Perot cavity of a crystal laser was replaced by a scattering surface^25^. Nonetheless, the RL coherent behavior, represented by the presence of randomly distributed spikes superimposed to the emission spectra, was only observed about thirty years later^26^. As the scattering elements are spatially separated from the active medium, this architecture has been conceptualized as relying on a spatially localized feedback mechanism^23,24^.

Even though this mechanism has been demonstrated in polymeric films, it has not been shown to occur in on-chip integrated microstructures. Thus, in this paper, we demonstrate low threshold lasing action in dye-doped polymeric microstructures based on diffuse reflections at the structure sidewall surface as spatially localized feedback. In the lasing architecture described here, the microstructures volume serves as an amplifying medium while the sidewall surfaces act as back-scattering reflectors and output couplers. The microstructures were produced by means of femtosecond laser writing via two-photon polymerization^27,28^, a technique that exploits the nonlinear nature of the two-photon absorption phenomenon to fabricate high-resolution 3D microstructures.

## Results and Discussions

**Figure 1:**
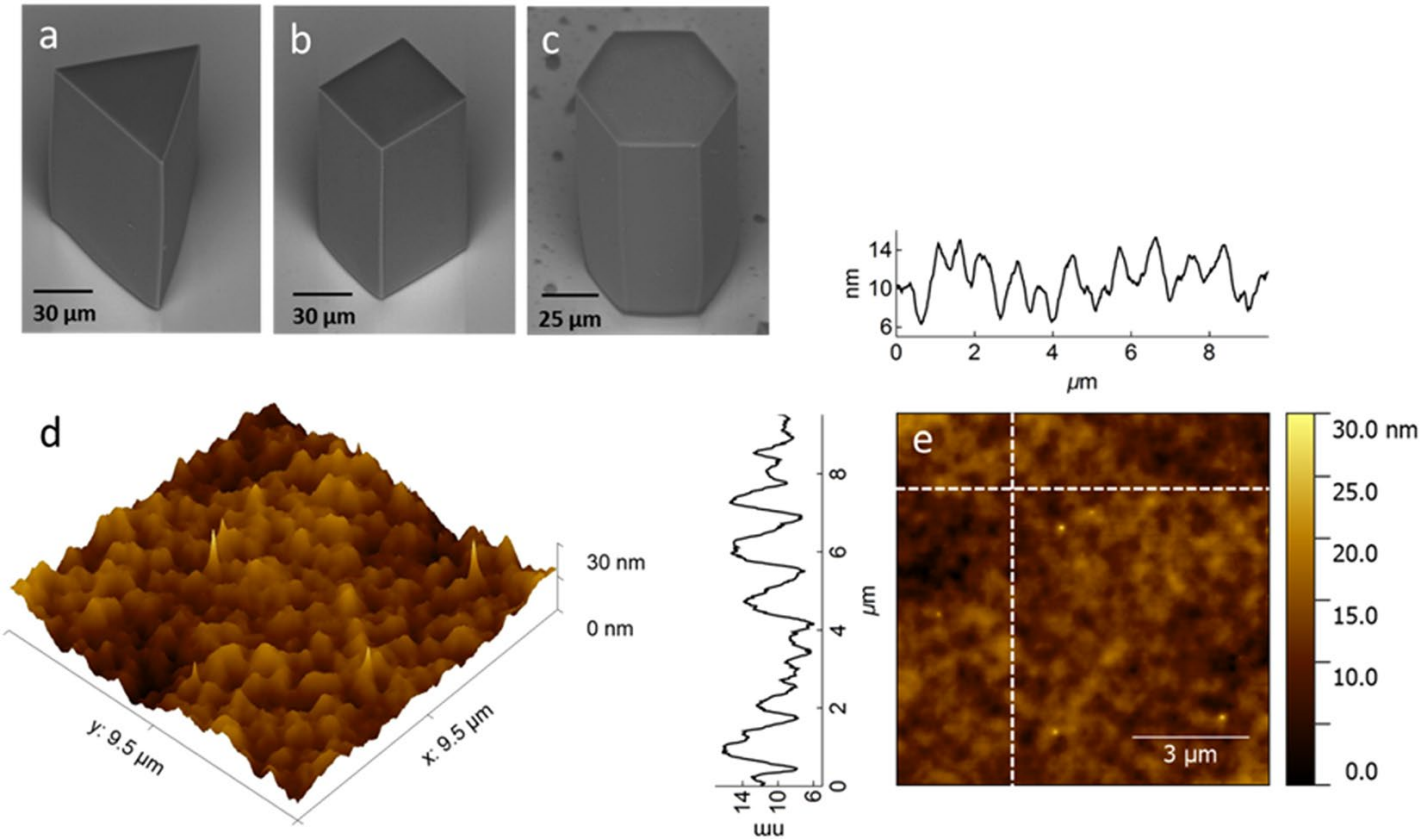
(**a**–**c**) Scanning electron micrographs of Rhodamine B-doped polygon-shaped microstructures fabricated by two-photon polymerization. (**d**) Atomic force micrograph of the sidewall surface of a microcube. (**e**) The same atomic force micrograph as depicted in the item d represented in a 2D chart. Two profile lines (indicated in the chart) are shown.

The dye-doped polymeric microstructures were fabricated by employing 0.1 nJ pulses from a Ti:sapphire oscillator with 40 µm/s of laser scan speed. As depicted in Fig. 1a–c, they are polygon-shaped structures featuring good structural quality and low shrinkage. They were designed in such a way that they all have the same volume, thus the triangle, cube, and hexagon exhibit edges of 76, 50, and 31 µm, respectively, whereas their height is 115 µm. By carrying out atomic force microscopy, the morphology of the microstructures sidewall surface was characterized and their average roughness was found to be 2 nm (Fig. 1d). Even though the sidewall average roughness is low, the size of the morphology features is comparable to the wavelength of the light emitted by the active medium. These irregularities in the refractive index on the surface thus act as back-scattering reflectors, providing positive feedback for random lasing. Figure 1e shows morphology profile lines from which the size of the morphology features can be better visualized. The active material is Rhodamine B (RhB), which is homogeneously distributed throughout the structures^7^.

Each micropolygon was pumped from above with a 100-ps pulsed laser operating at 532 nm, producing a Gaussian intensity profile over the structure top surface. As the beam spot is larger than the structures dimensions, they were entirely illuminated. Pulsed excitation prevents population transfer to the triplet lowest state, which would otherwise drop the fluorescence quantum yield of the dye^2^. Besides, given that the fluorescence lifetime of Rhodamine B is on the order of a few nanoseconds^29^, the pump carried out in the picosecond regime stands for less molecular cycles, thus extending the device lifetime.

**Figure 2:**
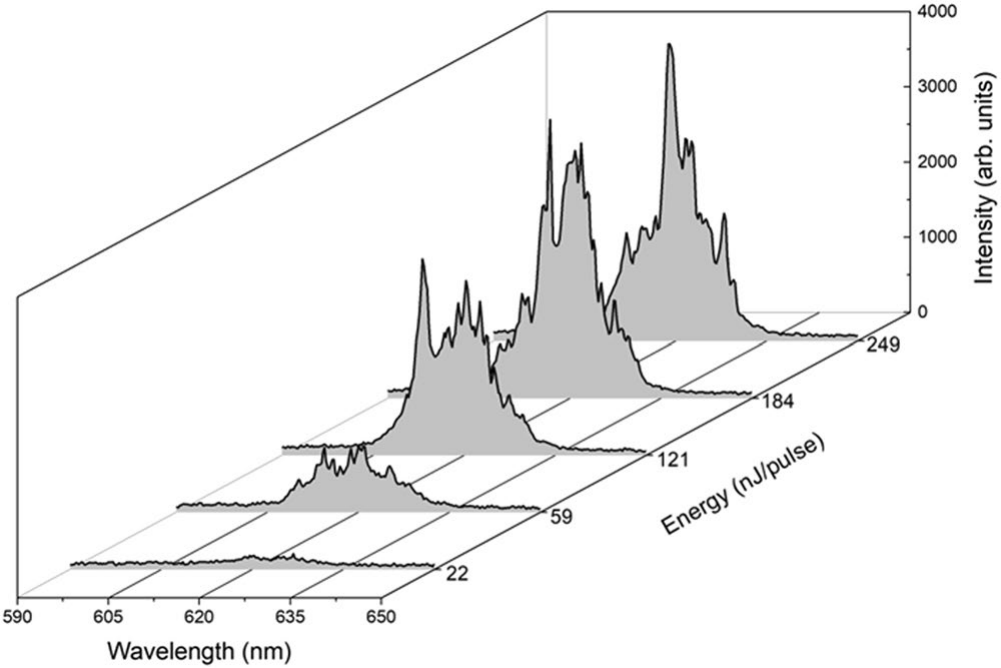
Emission spectra of a Rhodamine B-doped microcube for different pump energy levels. The first curve represents the spectrum taken around lasing threshold.

Figure 2 shows emission spectra collected for a microcube at different pump energies. As the pump energy is increased, a set of sharp peaks at random wavelengths appears, being superimposed to the Lorentzian-shape emission curve with maximum at 620 nm and 20 nm of full width at half maximum (FWHM). Notwithstanding the broadband emission of RhB that extends from 550 to 700 nm, the peaks only show up within a narrow spectral window, indicating the onset of laser action. The multimode behavior of emission with no evident spectral periodicity is a clear signature of resonant random laser and has been observed for all the polygon-shaped structures.

**Figure 3:**
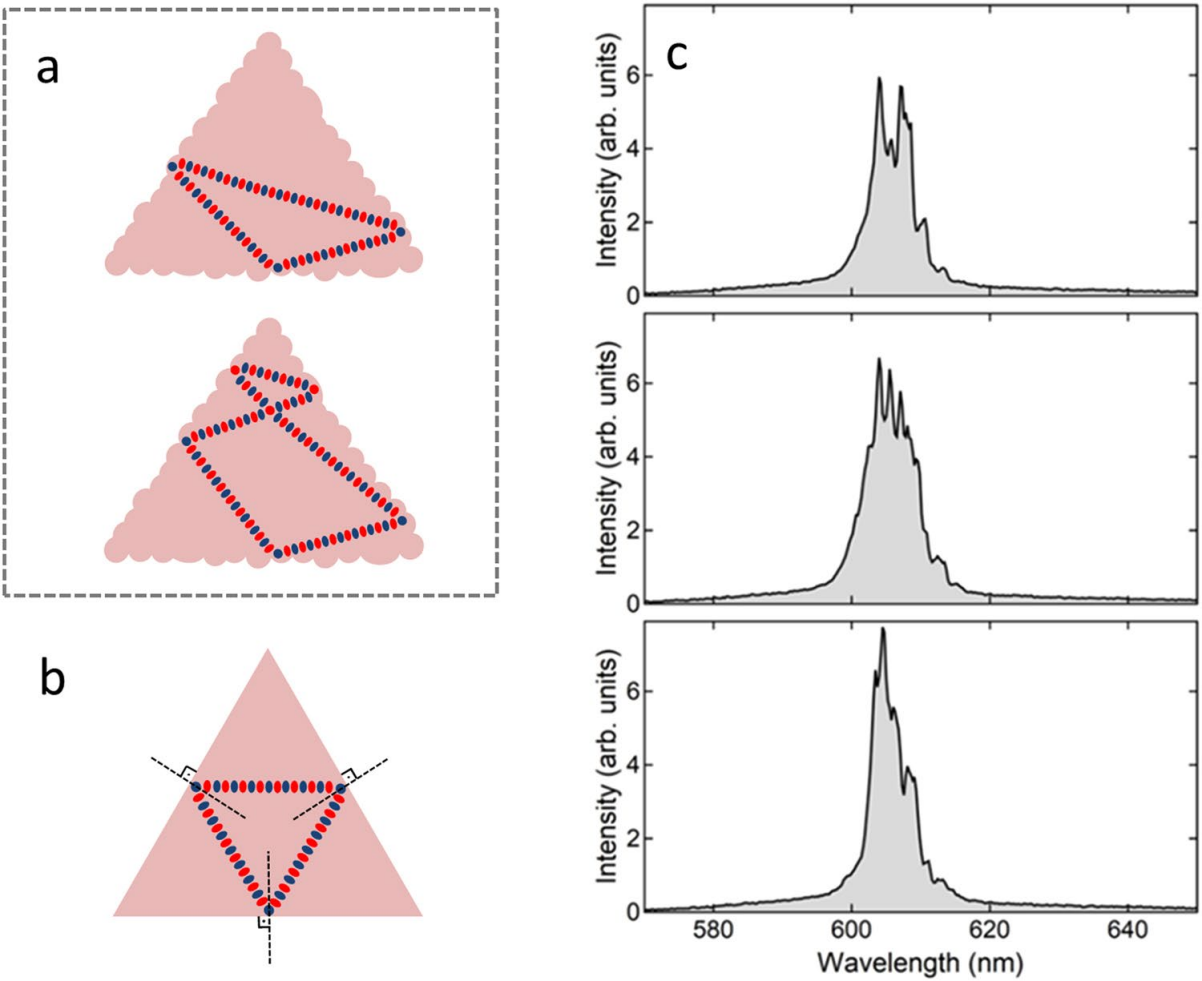
Top view schemes of feedback loops that can be formed by (**a**) diffuse reflections and (**b**) specular reflections in a triangular-shaped microstructure. (**c**) Emission spectra of a Rhodamine B-doped triangle-shaped microstructure taken for the same pump energy level (165 nJ/pulse).

The peaks are a result of successive diffuse reflections of light at the structure sidewall surfaces that re-enters the gain medium and closes feedback loops with different perimeters and Q-factors in the microstructure volume. Thus, the volume serves as an amplifying medium while the sidewall surfaces act as back-scattering reflectors and output couplers. Some examples of feedback loops that may be formed by diffuse reflections in a triangular-shaped structure are illustrated in Fig. 3a. Additionally, in a previous work^7^ we have shown that a hollow dye-doped microcube does not exhibit lasing when excited with the same pump energy levels employed to excite the filled polygon-shaped microstructures presented here, further confirming that the emitted light propagates in the volume.

The fact that no spectral periodicity is observed in the emission spectra rules out the contribution of specular reflections to lasing feedback. Specular reflections would otherwise give rise to fixed feedback loops inside the structure, leading to a set of either evenly spaced and well-defined peaks in the emission spectra. For example, the only possible feedback loop formed by specular reflections in the triangular-shaped structure would be an equilateral triangle loop (Fig. 3b), though no signature of this “cavity” was observed in the structure emission spectra collected for the same pump energy (Fig. 3c).

**Figure 4:**
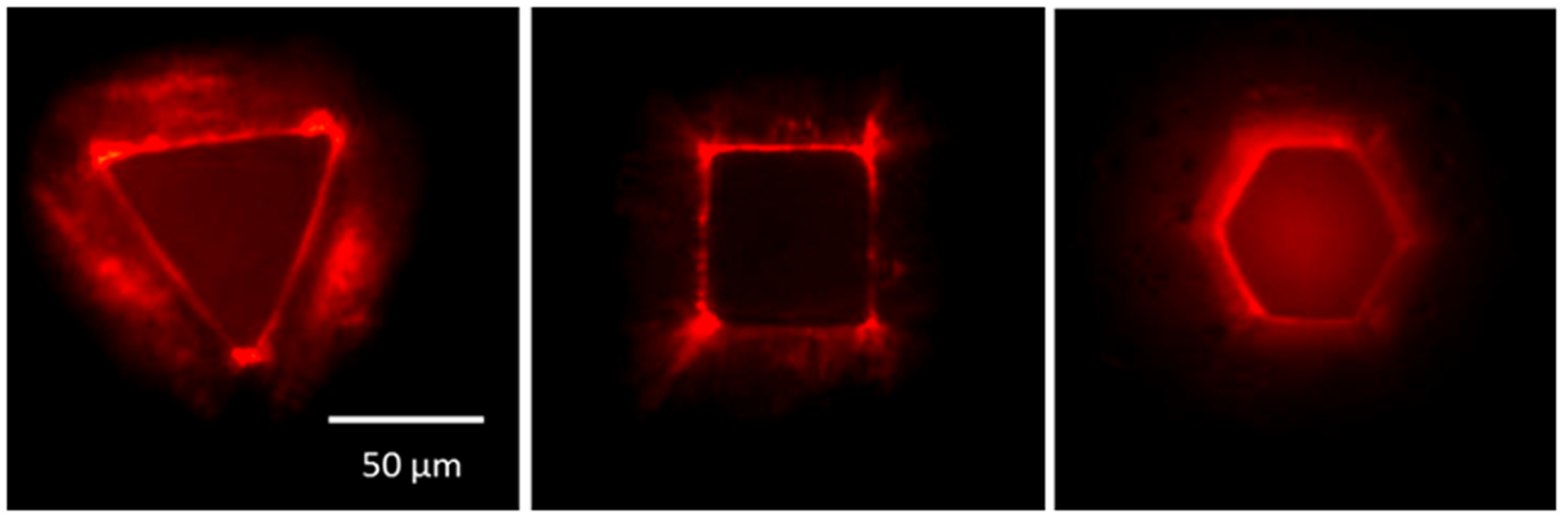
Top-view optical transmission images of a (**a**) triangle-, (**b**) cubic- and (**c**) hexagon-shaped microstructures under excitation at 165, 120 and 170 nJ/pulse, respectively. A color filter was added to the setup to block the excitation light.

The crosslinked polymer may act as scattering medium itself, which combined with the presence of the dye, leads to random lasing action via spatially distributed feedback. However, there was no evidence of distributed feedback due to scattering at imperfections spread over the volume of the microstructures. As can be seen in the images of the micropolygons being excited at energy levels above lasing threshold (Fig. 4), only fluorescence was observed coming out of the volume of the structures. Conversely, a strong emission was observed in the edges, indicating the presence of scattering elements in the microstructures sidewall surface. They are mostly spread along the structure perimeters due to the presence of protuberances in the corners. It is important to mention that we have followed a procedure to improve the surface quality of the microstructures top surface so as to prevent scattering coming out from it. This procedure is described in detail in the experimental section.

**Figure 5:**
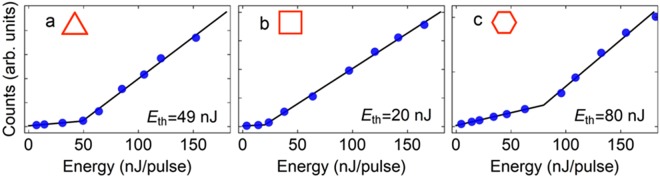
Integrated emission intensity as a function of pump energy for (**a**) triangle-, (**b**) cubic- and (**c**) hexagon-shaped microstructures (threshold energies are shown in each corresponding graph).

The behavior of the emitted light as a function of the pump energy was obtained by integrating the emission intensity over the lasing spectrum for several pump energy levels. The obtained results are illustrated in Fig. 5. A clear lasing threshold was measured at approximately 50, 20 and 80 nJ of pulse energy for the triangle-, cubic- and hexagon-shaped structures, respectively, by fitting the emission dependence on the pump energy to a bilinear curve. Such threshold energies are comparable to which have been achieved for polymer microlasers relying on usual cavities^7,8,30,31^.

## Conclusion

We reported on random lasing in dye-doped polymeric microstructures fabricated by means of femtosecond laser writing via two-photon polymerization. Randomly distributed irregularities in the microstructures sidewall surface act as back-scattering elements, providing positive feedback for lasing. The optically active material is Rhodamine B, which is homogeneously embedded in the polymer matrix. As the gain medium is spatially separated from the feedback elements, the pump is efficiently absorbed. This represents a great advantage over most of the random lasing devices, in which most of the pump light is scattered instead of being absorbed by the gain material.

A multimode emission with no evident spectral periodicity was obtained for free-space pulsed excitation of the structures at 532 nm. This behavior, which was systematic for all the geometries analyzed, is a clear signature of resonant random laser. The lasing threshold was found to be comparable to what have been reported for polymer microlasers, whose feedback relies on usual cavities. These results thereby show that these devices hold great potential as on-chip solid-state organic lasers.

## Methods

The microstructures were fabricated using a negative-tone photoresist composed of two acrylate monomers and a photoinitiator^32^. The monomers *tris* (*2-hydroxy ethyl*) *isocyanurate tryacrylate* (*SR368 – Sartomer*^®^) and *dipentaerythritol pentaacrylate* (SR399 - *Sartomer*^®^) were mixed in a proportion of 10/90 wt%. As photoinitiator we chose the *2*,*4*,*6-trimethylbenzoyl phenyl phosphinate*, an acylphosphine oxide photoinitiator commercially known as Lucirin TPO-L (in excess of 3 wt%, *Irgarcure*^®^). Rhodamine B (*Sigma-Aldrich*^®^) was first dissolved in ethanol and easily incorporated to the polymeric resin in a concentration of 10 µmol/g of resin. The solution was mixed for half an hour and left to rest by 48 hours. Once the solvent had completely evaporated, a drop of the photoresist was sandwiched between a glass substrate and a cover slip separated from each other with a 115 µm spacer. The sample was placed on a translation stage mounted on an inverted microscope. 100-fs pulses of a mode-locked Ti:sapphire oscillator (86 MHz repetition rate) operating at 780 nm were focused into the volume of the polymeric resin using a NA 0.25 objective lens. 3D microfabrication was performed by controlling both a galvanometric-mirror system and the stage that supports the sample with a computer-aided software. A detailed description of the microfabrication process can be found elsewhere^33^. The microstructures shown herein were fabricated layer by layer until they reach the cover slip, thus their height is limited by the spacer placed between the glass substrate and the cover slip. Besides setting the microstructures top surface notably flat, this strategy helps to reduce the top surface roughness by making it reproduce the roughness of the glass surface.

The setup assembled for measuring the emission spectra of the microstructures used as excitation source a frequency-doubled (532 nm) Q-switched/mode-locked Nd:YAG laser operating at 100 Hz of repetition rate, which delivers a sequence of 100-ps pulses modulated by the Q-switched envelope (pulse train). To set the excitation to single pulse operation, a Pockels cell and a polarizer were added to the system. The laser beam was loosely focused on the microstructure top surface, resulting in a beam waist with 100 µm of radius. Microstructure emission was acquired by positioning a multimode optical fiber in the proximity of the structure sidewall surface and connecting its other end to a spectrometer (*Ocean Optics HR4000*^®^). The substrate containing the microstructures rested on an inverted microscope coupled to a CCD camera that allows real time monitoring of the excitation process. A half-wave plate, combined with a polarizer, was used to tune the energy delivered to the structures.

The microstructures were characterized by scanning electron microscopy (*Hitachi TM3000*^®^) and their surface quality was obtained by directly measuring their sidewall roughness with atomic force microscopy (*Nanosurf FlexAFM*^®^).

## Data Availability

All data generated or analyzed during this study is included in this published article.
